# Lactoferrin as a multi-target nutritional modulator in type-2 diabetes

**DOI:** 10.3389/fnut.2026.1833440

**Published:** 2026-07-02

**Authors:** Yalçın Mert Yalçıntaş, Sercan Karav, Duygu Aǧagündüz

**Affiliations:** 1 Department of Molecular Biology and Genetics, Canakkale Onsekiz Mart University, Canakkale, Türkiye; 2 Department of Nutrition and Dietetics, Gazi University, Ankara, Türkiye

**Keywords:** diabetes, lactoferrin, metabolic, modulator, nutrition

## 1 Introduction

Diabetes mellitus is a metabolic disorder that is marked by persistently high blood glucose levels, caused by disturbances in the secretion or function of the hormone insulin (1). Several types of diabetes mellitus have been described: Type 1 diabetes, a type of diabetes that occurs as a consequence of the autoimmune destruction of β-cells in the pancreas, and typically manifests in early life; Type 2 diabetes, the most prevalent form of diabetes, in which there is a combination of insulin resistance and relative insulin deficiency; and gestational diabetes, a type of diabetes that manifests during pregnancy and typically resolves after childbirth (2). Among these conditions, type 2 diabetes has gained increasing attention because of its rising incidence and multifactorial pathophysiology. It is now recognized not only as a disorder of glucose metabolism but also as a condition closely associated with immunometabolic dysfunction, in which inflammation, immune regulation, and metabolic homeostasis are interconnected (3).

The complex and multifactorial nature of type 2 diabetes limits the effectiveness of approaches that focus on a single biological target (4). Consequently, in recent years, there has been growing interest in multi-target nutritional approaches that can simultaneously target inflammation, gut barrier function, and metabolic pathway signaling (5–7). Bioactive proteins and dairy extracts, as a functional nutrition-related category, are highlighted as a supportive strategy in the treatment of metabolic disorders. In this multi-targeted nutritional strategy, Lactoferrin (Lf) is highlighted as a multifunctional glycoprotein with iron-binding affinity, predominantly found in high concentrations in milk and colostrum (8). Because of its anti-inflammatory properties, immune response regulation ability, and potential role in the gut environment, Lf is introduced as a bioactive compound that needs to be re-evaluated in the context of the immunometabolic concept of type 2 diabetes. In this perspective, considering the immune-metabolic effects of Lf on inflammation, together with its interactions with the integrity of the gut barrier and microbiota, it becomes feasible to better understand how it can be placed within multi-targeted nutritional strategies. Considering the relationships between the pathways of inflammation, metabolic stress originating from the gut, and energy homeostasis, it is necessary to assess Lf not only as a complementary factor but also as a potential regulator that may modulate the immune-gut-metabolic axis in the pathophysiology of type 2 diabetes. The aim of this opinion piece is to examine the potential effects of Lf in the context of type 2 diabetes from the perspectives of inflammation, gut integrity, and metabolic signaling pathways, and to discuss its potential role in functional nutrition strategies.

## 2 Lactoferrin in the immunometabolic axis of type-2 diabetes

Lf is considered a glycoprotein that may exert its influence on the regulation of immune-metabolic inflammation through multiple biological pathways (9). Chronic low-grade inflammation observed in type 2 diabetes is particularly associated with activation of the nuclear factor kappa B (NF-κB) signaling pathway, increased pro-inflammatory cytokine production, and alterations in macrophage polarization (10, 11). Due to its iron-binding capacity, Lf may limit free iron-induced oxidative stress, which may contribute to the suppression of inflammatory signals by reducing the production of reactive oxygen species (ROS) (12). Furthermore, some experimental studies suggest that Lf can reduce M1 phenotype-directed pro-inflammatory macrophage activity through its effects on immune cells, thus indirectly modulating adipose tissue inflammation (13). These processes provide a possible mechanistic framework through which Lf may support insulin receptor signaling; however, direct confirmation in humans remains limited. Therefore, Lf can be considered a potential regulator that may influence the immune-metabolic axis, which is involved at the intersection of inflammation, oxidative stress, and immune responses.

In the pathophysiology of type 2 diabetes, disruption of intestinal barrier integrity and changes in microbiota composition are closely associated with metabolic endotoxemia and increased systemic inflammation (14). An increased intestinal permeability may result in the translocation of bacterial components, such as lipopolysaccharides (LPS), into the bloodstream and the subsequent activation of Toll-like receptor (TLR)-mediated inflammatory pathways that contribute to insulin resistance (15). Lf is a molecule that has been identified as a potential factor in maintaining the balance of gut microbiota, primarily owing to its iron-binding activity and its ability to selectively modulate microbial growth (16). The experimental evidence indicates that Lf has the ability to maintain epithelial cell integrity and tight junction proteins, which may open a new avenue for the attenuation of gut-derived inflammation (17). In this context, Lf has been identified as not only an antimicrobial protein but also a potential regulator molecule that may target the gut-immune-metabolic axis.

**Figure 1:**
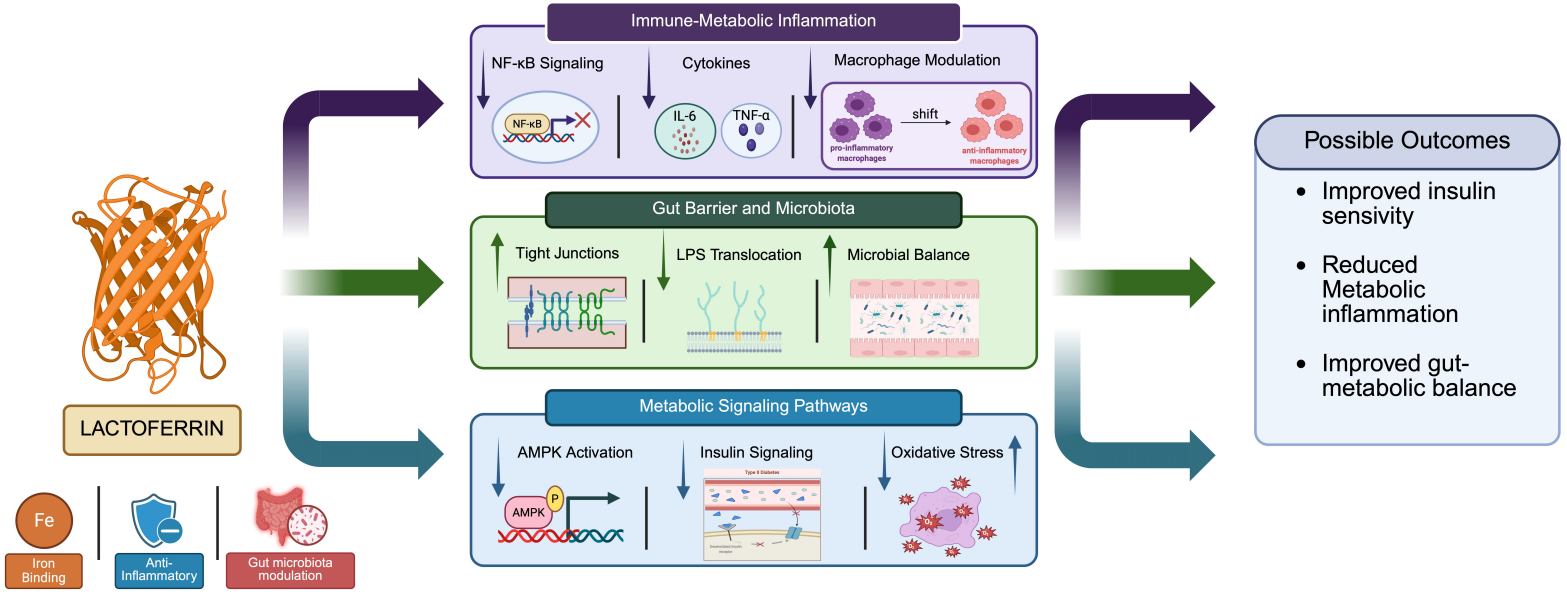
Proposed multi-target mechanisms of lactoferrin in type-2 diabetes. Created with BioRender.com.

Aside from its immune regulatory properties and intestinal barrier function, Lf is also regarded as a bioactive protein that is connected to the signaling pathways involved in insulin sensitivity and cellular metabolic regulation. Experimental evidence suggests that Lf could potentially affect the metabolic regulatory pathways such as AMP-activated protein kinase (AMPK) that are involved in glucose uptake, lipid metabolism, and energy homeostasis (18, 19). The decrease in oxidative stress and inflammatory signaling could potentially create a biological milieu that plays a role in the maintenance of insulin receptor signaling. Therefore, Lf should be considered not as a direct glucose-lowering agent, but as a supportive regulator of metabolic function (Figure 1).

From the perspective of functional nutrition, Lf is a bioactive compound that can be used in a nutritional approach aimed at multiple objectives, including the reduction of inflammation, the maintenance of the integrity of the intestinal barrier, and the modulation of metabolic signaling pathways. Instead of being a substitute for pharmacological therapies, the use of functional compounds extracted from milk or colostrum containing Lf can be seen as part of a nutritional approach aimed at the maintenance of metabolic health. Bioavailability and dosing remain important limitations for the nutritional use of Lf. Orally administered Lf may be partially degraded during gastrointestinal digestion, and its activity may depend on both intact Lf and digestion-derived peptides. In addition, bovine and human Lf differ in structure and glycosylation, which may influence stability and biological activity. Therefore, delivery strategies such as enteric-coated formulations, encapsulation, or nanoparticle-based systems may be useful to improve gastrointestinal stability. Current human studies have used fixed oral doses, such as 250–300 mg/day, but the optimal dose, formulation, and duration of Lf supplementation in type 2 diabetes remain unclear (20, 21).

Although the biological plausibility of LF in type 2 diabetes is mainly supported by *in vitro* and animal studies, the available human evidence should also be considered. Experimental studies have provided mechanistic support for the effects of Lf on inflammation, oxidative stress, gut barrier integrity, and metabolic signaling pathways (12, 13, 18, 19). Observational human studies have reported associations between circulating Lf levels, altered glucose tolerance, insulin resistance, obesity-related parameters, and inflammatory biomarkers, suggesting a possible link between Lf and metabolic dysfunction (9, 20). In addition, a clinical intervention study in obese pediatric patients with type 2 diabetes reported that oral Lf supplementation improved glycemic, inflammatory, oxidative stress-related, and metabolic parameters (22). However, current clinical evidence remains limited, and most available intervention data are derived from small or specific patient populations. Therefore, well-designed randomized controlled trials in adults with type 2 diabetes are required to clarify the dose-response relationship, bioavailability, long-term safety, and clinical efficacy of Lf supplementation.

A balanced interpretation of these findings is necessary because the current evidence base has several limitations. First, many proposed mechanisms of Lf action are derived from *in vitro* studies and animal models, and these findings cannot be directly translated to clinical outcomes in humans. Second, available observational studies can demonstrate associations between Lf-related parameters and metabolic dysfunction, but they do not establish causality. Third, clinical intervention data remain limited in number, sample size, population diversity, and follow-up duration. Differences in study design, participant characteristics, Lf source, dose, formulation, and outcome measures also make direct comparison between studies difficult. Therefore, while Lf represents a biologically plausible nutritional candidate for supporting metabolic health, its clinical applicability in type 2 diabetes remains uncertain and should be interpreted cautiously until larger, well-controlled human trials are available.

## 3 Conclusion

This opinion article stresses that the assessment of Lf in the pathophysiology of type 2 diabetes should not be focused on a single mechanism but on several biological processes, such as the regulation of inflammation, the maintenance of intestinal barrier function, and its interaction with metabolic pathways. The potential regulatory function of Lf in the immune-gut-metabolic axis supports its further evaluation as a functional nutrition component. Although the current data do not yet allow for the establishment of conclusive clinical results, the multi-targeted action of Lf represents a major field of discussion for the development of supportive nutritional therapies in complex metabolic disorders such as type 2 diabetes.

To better explain the possible role of Lf in the context of type 2 diabetes, experimental research is required to confirm the specific signaling pathways at the mechanistic level. In this regard, a holistic strategy to assess the relationship between intestinal barrier function, inflammation, and metabolic signaling pathways may provide new insights into the role of lactoferrin in the immune-gut-metabolic axis. Moreover, clinical research studies focusing on the differences in dose ranges, bioavailability, and individual differences in responses are essential for applying the current mechanistic knowledge to nutritional practices. In the future, the integration of multi-omics analysis and microbiota studies may help to identify the potential role of lactoferrin in the context of personalized nutritional approaches. In this regard, the assessment of Lf together with functional components from colostrum and milk may provide new avenues for research in the development of multi-targeted nutritional approaches.
